# Chest X-ray Severity Score as a Putative Predictor of Clinical Outcome in Hospitalized Patients: An Experience From a Vietnamese COVID-19 Field Hospital

**DOI:** 10.7759/cureus.23323

**Published:** 2022-03-19

**Authors:** Sy Van Hoang, Kha Minh Nguyen, Tien Manh Huynh, Khoa Le Anh Huynh, Phong Hoai Nguyen, Hai Phuong Nguyen Tran

**Affiliations:** 1 Department of Internal Medicine, University of Medicine and Pharmacy at Ho Chi Minh City, Ho Chi Minh, VNM; 2 Department of Biostatistics, Virginia Commonwealth University School of Medicine, Richmond, USA; 3 Department of Pediatrics, University of Medicine and Pharmacy at Ho Chi Minh City, Ho Chi Minh, VNM; 4 Interventional Cardiology, Cho Ray Hospital, Ho Chi Minh, VNM

**Keywords:** prediction, chest x-rays, covid-19, tss score, brixia score

## Abstract

Background

Through the coronavirus disease 2019 (COVID-19) pandemic, portable radiography was particularly useful for assessing and monitoring the COVID-19 disease in Vietnamese field hospitals. It provides a convenient and precise picture of the progression of the disease. The purpose of this study was to evaluate the predictive value of chest radiograph reporting systems (Brixia and total severity score (TSS)) and the National Early Warning Score (NEWS) clinical score in a group of hospitalized patients with COVID-19.

Methods

This retrospective cohort study used routinely collected clinical data from polymerase chain reaction (PCR)-positive severe acute respiratory syndrome coronavirus 2 (SARS-CoV-2) patients admitted to Field Hospital District 8, Ho Chi Minh City, Vietnam, from August 2021 to September 2021. The initial chest radiographs were scored based on the TSS and Brixia scoring systems to quantify the extent of lung involvement. After the chest radiograph score was reported, two residents calculated the rate of all-cause in-hospital mortality with the consultation of expert radiologists. In this study, NEWS2 scores on hospital admission were calculated. The gradient boosting machines (GBMs) and Shapley additive exPlanations (SHAP) were applied to access the important variable and improve the accuracy of mortality prediction. The adjusted odds ratio for predictor was presented by univariate analysis and multivariate analysis.

Results

The chest X-rays (CXRs) at the admission of 273 patients (mean age 59 years +/-16, 42.1% were male) were scored. In the univariate analysis, age, vaccination status, previous disease, NEWS2, a saturation of peripheral oxygen (Sp02), the Brixia and TSS scores were signiﬁcant predictors of mortality (p-value < 0.05). In multivariate analysis, there were statistically significant differences in mortality between age, Sp02, Brixia score, and patients with previous diseases were independent predictors of mortality and hospitalization.

A gradient boosting machine was performed in the train data set, which showed that the best hyperparameters for predicting the mortality of patients are the Brixia score (exclude TSS score). In the top five predictors, an increase in Brixia, age, and BMI increased the logarithmic number of probability clarifying as death status. Although the TSS and Brixia scores evaluated chest imaging, the TSS score was not essential as the Brixia score (rank 6/11). It was clear that the BMI and NEWS2 score was positively correlated with the Brixia score, and age did not affect this correlation. Meanwhile, we did not find any trend between the TSS score versus BMI and NEWS2.

Conclusion

When integrated with the BMI and NEWS2 clinical classification systems, the severity score of COVID-19 chest radiographs, particularly the Brixia score, was an excellent predictor of all-cause in-hospital mortality.

## Introduction

In March 2020, the World Health Organization (WHO) declared coronavirus disease 2019 (COVID-19) as a global pandemic [1]. Vietnam was a spectacular COVID-19 success story, reporting zero cases for months on end and maintaining life as normal as possible for the majority of the population. However, in late April 2021, the highly transmissible Delta variant began to spread across Vietnam. Especially, in the fourth wave of this pandemic, Ho Chi Minh City, the country's economic engine, where 13 million people live and work, became the epicenter of the virus's battle: hundreds of cases are documented daily amid extensive testing [2]. As of August 2021, there were over 200,000 cases in the community of Ho Chi Minh City alone, with another 100,000 cases in other southern and central provinces [2]. Several primary hospital wards were reorganized and exclusively dedicated to patients with COVID-19. The emergency department (ED) of the field hospital was overflowing with patients afflicted by COVID-19 symptoms; thus, providing adequate stratification was a life-saving urgency. In addition, the severe acute respiratory syndrome coronavirus 2 (SARS-CoV-2) infection rapidly changes, leading to an increase in confirmed cases in Vietnam. The progression of lung aberrations in COVID-19 patients requires fast radiological methods and appropriate treatment for infected patients. CT imaging was the best method of assessing the specific abnormalities of the disease in developed countries [1,3-5]. However, the increasing number of hospitalized patients, lack of experienced radiologists, risk of cross-over infection, high cost, and increased radiological examinations make it challenging to use chest CT [3-5]. Furthermore, due to its usefulness, convenience, wide availability, and ease of performance at the bedside, several studies demonstrated that CXR is a non-inferior, first-line triage substitute to diagnose COVID-19 and determine the severity as well as the progression of respiratory conditions [3,5-6]. In addition, the interpretation of portable CXRs has always been utilized in medical fields to diagnose the causes of respiratory problems.

Various objective measures have been applied to reduce the wide variability between observers in assessing lung involvement. Many descriptive reporting semi-quantitative scoring systems, such as Brixia and total severity score (TSS) on the severity of lung disease, were adopted for triage in ED combined with clinical data and to classify lung lesions, which helped assess the lung injury and correlated with adverse outcomes [5,7-16]. The Brixia scoring system has been widely used to monitor the severity and progression of COVID-19 pneumonia in Tongji Hospital, Wuhan, and Azienda Socio Sanitaria Territoriale, Spedali Civili of Brescia, Italy [8-9]. This study aimed to find the best chest X-ray (CXR) score associated with the mortality of COVID-19 patients. TSS is a system presented by Wong et al. in “Radiology” in March 2020 [11]. The authors adapted and simplified the Radiographic Assessment of Lung (O)Edema (RALE) score proposed by Warren et al. in 2018 [10]. The Brixia score is another method to assess CXR, explicitly designed for patients with confirmed COVID-19 [8-9]. The Vietnamese Ministry of Health recommended the TSS to evaluate COVID-19 patient CXRs [9]. Borghesi and Maroldi proposed it in March 2020 [8]. One of the most significant differences between the two scoring systems is that a general practitioner can perform TSS due to its ease of use. At the same time, the Brixia score is intended for use by a professional radiologist [6,8,10]. Given the current conditions regarding the lack of distribution of resources in primary healthcare centers and the need for early stratification to optimize individualized treatment, non-radiologist clinicians have the responsibility to evaluate the portable CXR and make a final clinical decision. The purpose of this study is to determine the clinical utility of scoring systems through portable CXR in Vietnamese field hospitals and explore their potential hospitalized prediction of COVD-19 patient mortality.

## Materials and methods

Study design and participants

**Figure 1:**
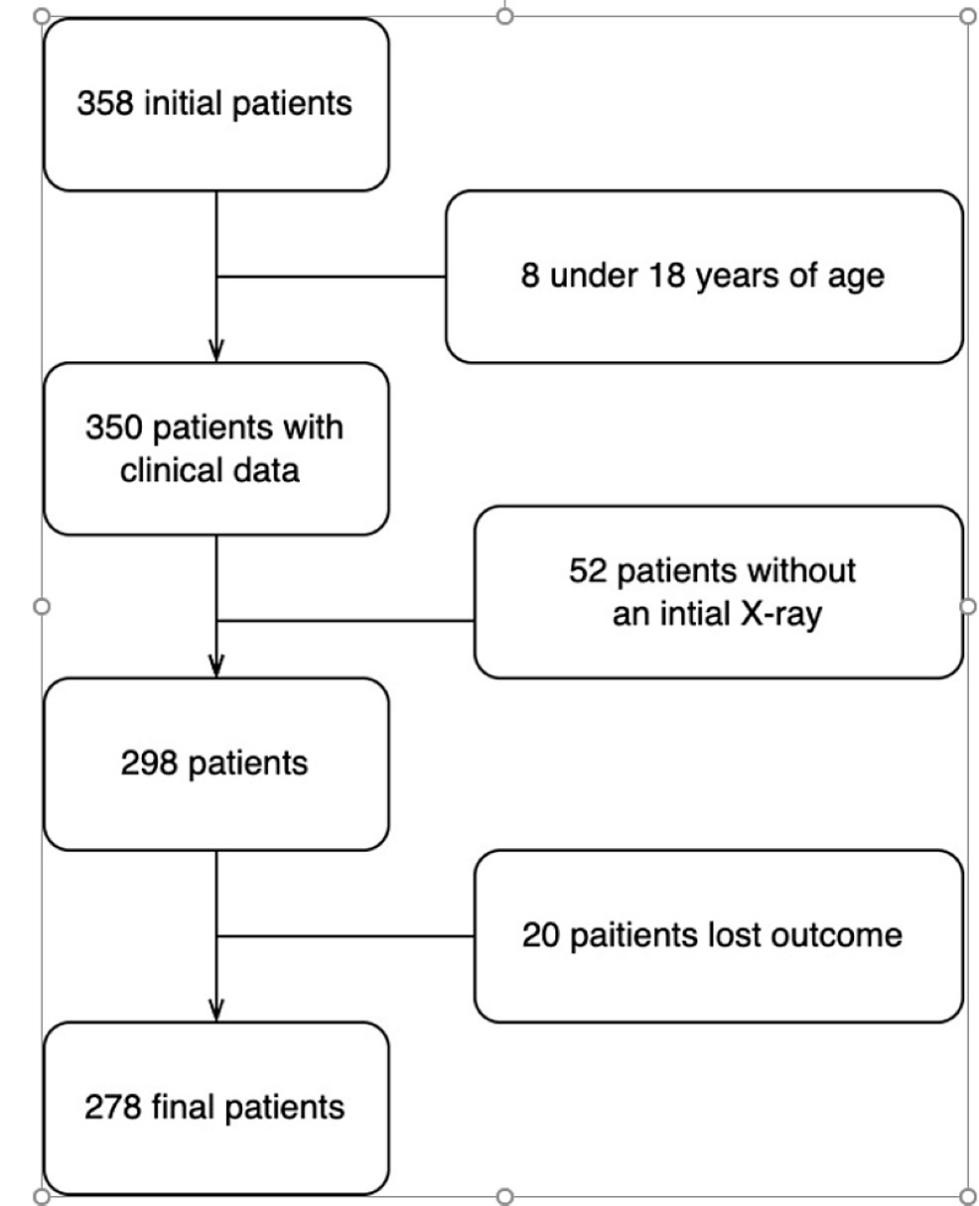
Flow chart of study design

A descriptive, cross-sectional, retrospective, single-center study was conducted between August 2021 and September 2021. We randomly selected patients based on: (a) initial portable CXR performed in the ED, (b) positive reverse transcription-polymerase chain reaction (RT-PCR) test, and (c) and mortality (discharge or death). However, we excluded patients: (a) for whom we could not collect clinical data or (b) had non-diagnostic CXR image quality. A total of 278 patients were selected in our study (Figure 1).

Patient demographics and clinical data collection

The demographic variables collected included age and gender. Additional clinical variables included past medical history, body mass index (BMI), smoking history, comorbidities, RT-PCR results, vital signs, oxygen saturation (SpO2), and outcome. The mentioned clinical data were obtained from medical records.

Imaging data collection

Portable chest X-rays were obtained using the following equipment: DRGEM - Jade (DRGEM Corporation, South Korea). In this study, the initial CXR at the emergency department was analyzed. During the early stage of the pandemic, the chest X-ray report was not uniform and did not have a specific scoring system to analyze. In the isolation wards, if the patient was able to maintain a standing position, all initial chest X-rays were performed in a single frontal projection with a posterior-anterior view. For a sitting or supine position, an anteroposterior view was acquired. In the remaining cases, an anteroposterior view was acquired in a sitting or supine position.

The chest X-rays were evaluated by two internal residents with experience in thoracic imaging (one and two years of experience, respectively); discrepancies in the images were resolved by consensus after consulting with two thoracic radiologists with 10 years of experience. The internal residents used a semi-quantitative score to quantify the extent of pulmonary involvement. The severity of imaging was calculated using the Brixia and TSS scoring systems.

The Brixia scoring system divided the lungs into six regions. Two lines split the lungs into six areas; the first line was drawn at the level of the inferior wall of the aortic arch and the second line at the level of the right inferior pulmonary vein. The reviewers rated each region from 0 to 3 based on the severity of the lesion. The score ranges from 0 to 18. Score 0 for no lung abnormalities, 1 for interstitial infiltrates, 2 for interstitial and alveolar enters (interstitial dominant), and 3 for interstitial and alveolar enters (alveolar dominant). Other findings, such as pleural effusion and pulmonary vessel enlargements, were not included in the Brixia scoring system.

The degree of disease severity was assessed using the severity score proposed by Warren et al. [10]. TSS divided the lung into four regions for each lung, left and right [10]. The maximum score for TSS was 8. Each lung was evaluated individually. Then, depending on the extent of involvement of any of the four, including consolidation, interstitial lesions, blurred nodules, or ground-glass opacity, a score of 0 to 4 points was given (0 - no involvement; 1 - less than 25%; 2 - 25% to 50%; 3 - 50% to 75%; 4 more than 75% involvement). The overall score was the sum of points from both lungs.

Statistical analysis

Baseline characteristics were used to predict the clinical outcomes of two groups (recovery versus death). Our predictor variables included the Brixia score, Sp02, age, body mass index (BMI), at least had one dose of vaccine (yes/no), NEWS2, smoking status (yes/no), hypertension (yes/no), gender (male/female), and previous disease (yes/no).

A predictive comparison of two groups was conducted using a t-test. Categorical comparisons between the groups were performed using Pearson statistics. Exact tests were used if the minimum expected cell frequency assumption (n=5) was violated. Multivariate and univariate logistic regression models were performed to calculate the predictor variables' odds ratio and 95% confidence interval (95% CI).

Our study used gradient boosting machines (GBMs) to predict the outcome. We used the implementations provided in the caret package in the R software (R Foundation for Statistical Computing, Vienna, Austria). Since we want to put as many patients in our train model to find the best accuracy, we randomly split them into the train (80%) and test (20%) data. The train data would find the optimal hyperparameters for machine learning algorithms. We determined the results using five-fold cross-validation and 10 resampling iterations. The best hyperparameters were selected based on their accuracy. Furthermore, the area under the curve (AUC), sensitivity, and specificity were calculated. We utilized Shapley Additive exPlanations (SHAP) to see how each predictor contributed to the machine learning that predicted the outcome [17-20]. Feature selection predictors must satisfy three essential properties: accuracy, consistency, and missingness [18-20]. SHAP values measure the influence of a predictor by comparing the predictions of the machine learning model with and without that predictor. The rank of significant predictors based on the SHAP value would be visualized. Furthermore, the confounding between Brixia score, BMI, and NEWS2 would be visualized using SHAP values and locally estimated scatterplot smoothing (LOESS) regression. Statistical significance considered when p-value < 0.05. All analysis was done using R software (version 4.1.0).

## Results

A total of 278 patients participated in our study. Of those COVID-19 patients, 117 (42.1%) patients were male. The median age was 59 years, with 47.8% of patients having one vaccine shot. The majority of patients had underlying conditions (72.7%) such as obesity (23 patients), type 2 diabetes (14 patients), and hypertension (148 patients). Table 1 showed that age, vaccination, NEWS2, Sp02, and Brixia score were statistically significant differences between living and death statuses. The mean of age, NEWS2, and Brixia score in the living group were lower as compared to other groups.

**Table 1:** Comparison of baseline characteristics between living status and death status

	Outcome = 0 (n=227)	Outcome = 1 (n=51)	p-value
Age	58.52 (13.9)	64.80 (12.8)	0.003
Gender			0.213
Male	100 (44.1)	17 (33.3)	
Female	127 (63.9)	34 (66.7)	
Vaccinate			0.032
Yes	116 (51.1)	17 (33.3)	
No	111 (48.9)	34 (66.7)	
BMI	24.4 (3.28)	25.2 (3.8)	0.158
Underlying diseases			0.058
Yes	159 (70.0)	43 (84.3)	
No	68 (30.0)	8 (15.7)	
Hypertension			0.675
Yes	119 (52.4)	29 (56.9)	
No	108 (47.6)	22 (43.1)	
Smoking			0.252
Yes	70 (30.8)	11 (21.6)	
No	157 (69.2)	40 (78.4)	
NEWS2	7.85 (2.95)	9.16 (2.21)	0.0030
Sp02	84.9 (9.7)	79.0 (9.4)	< 0.0001
Brixia score	11.65 (4.4)	15.37 (2.9)	< 0.0001
TSS score	13.74 (5.9)	16.59 (4.6)	0.0003

**Figure 2:**
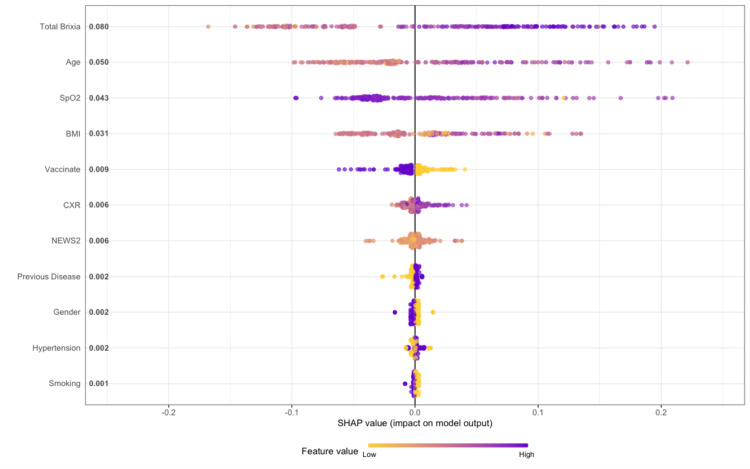
Ranking of important variables using Shapley Additive exPlanations

**Figure 3:**
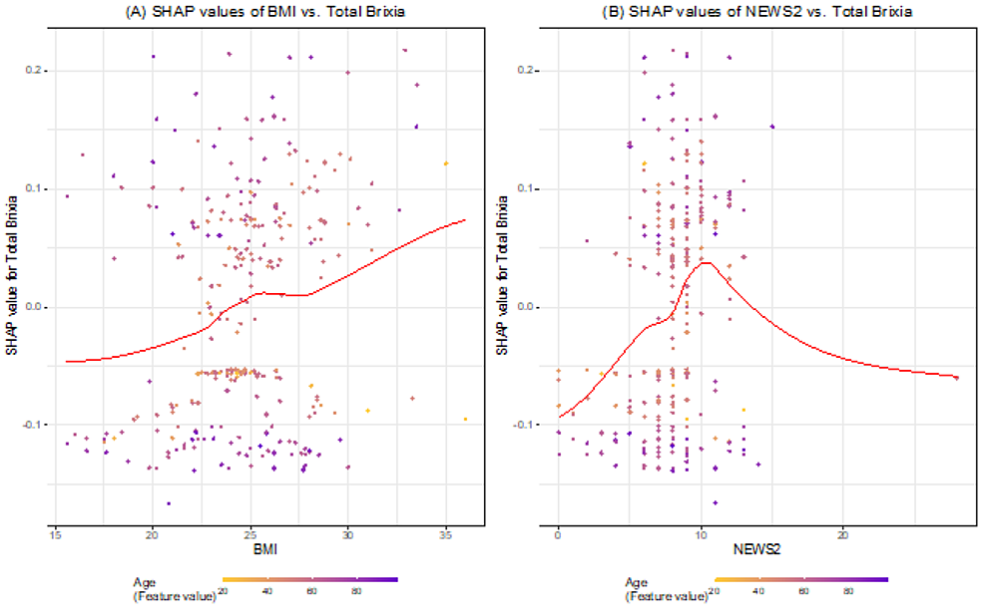
Shapley Additive exPlanations (SHAP) value of BMI and National Early Warning Score 2 (NEWS2) versus the Brixia score over age

Figure 2 shows the rank of important variables in predicting the outcome. We saw that the Brixia score was the most important feature compared to the others, with the mean SHAP value equal to 0.086. Each point represents patients in our data. The X-axis was the SHAP value, indicating how much was the change in log-odds of probability clarified as death status. Gradient color means the value for each predictor. In the top five predictors, increases in Brixia, age, and BMI led to an increase in log-odd of probability. Although the TSS score and Brixia score evaluated chest imaging, the TSS score was not as important as the Brixia score (rank 6/11). In Figure 3, it is clear that BMI and NEWS2 score were positively correlated with Brixia score, and age did not affect this correlation. However, we did not find any trend between TSS score versus BMI and NEWS2.

Table 2 showed univariate and multivariate logistics regression with odds ratios and corresponding 95% CI. On univariate analysis, age, vaccination, underlying conditions, NEWS2, Sp02, Brixia, and TSS were significant mortality predictors (p-value < 0.05). A patient who had a previous disease had the strongest association with mortality as compared to other predictors (OR = 2.29, 95% CI = 1.09 - 5.51, and p-value = 0.043). Age, NEWS2, and Brixia score were positively associated with an increase in mortality of 3%, 18%, and 30%, respectively. However, people with at least one vaccine and Sp02 were negatively associated with increased mortality. In multivariate analysis, there was a statistically significant difference in recovery rate between age, Sp02, total Brixia score, and patients who had a previous disease. Although the correlation between the total Brixia score and TSS score was 0.28 (p-value < 0.0001), we did not find any association between TSS and recovery rate.

**Table 2:** Logistic regression analysis between two groups NEWS 2: National Early Warning Score 2; Sp02: oxygen saturation; TSS: total severity score

	Univariate analysis		Multivariate analysis	
Variable	Odd ratio (95% CI)	P-value	Odd ratio (95% CI)	P-value
Age	1.03 (1.01 – 1.06)	0.0040	1.05 (1.02 - 1.09)	0.0022
Male	0.64 (0.33 - 1.19)	0.1630	1.00 (0.34 - 2.85)	0.9983
Vaccine = yes	0.48 (0.25 - 0.89)	0.0235	0.50 (0.23 - 1.06)	0.0760
BMI	1.07 (0.98 - 1.17)	0.1583	1.04 (0.92 - 1.17)	0.5702
Underlying disease = yes	2.29 (1.08 - 5.51)	0.0430	3.96 (1.20 - 14.11)	0.0268
Hypertension = yes	1.19 (0.65 - 2.26)	0.5660	0.54 (0.29 - 1.43)	0.2114
Smoking = yes	0.62 (0.29 - 1.24)	0.1910	0.53 (1.59 - 1.70)	0.2824
NEWS2	1.18 (1.05 - 1.34)	0.0073	1.18 (0.99 - 1.39)	0.0583
Sp02	0.95 (0.91 - 0.98)	0.0008	0.95 (0.92 - 0.99)	0.0075
Brixia score	1.30 (1.18 - 1.45)	< 0.0001	1.26 (1.13 - 1.43)	< 0.0001
TSS score	1.10 (1.04 - 1.18)	0.0017	1.07 (1.00 - 1.15)	0.0588

**Figure 4:**
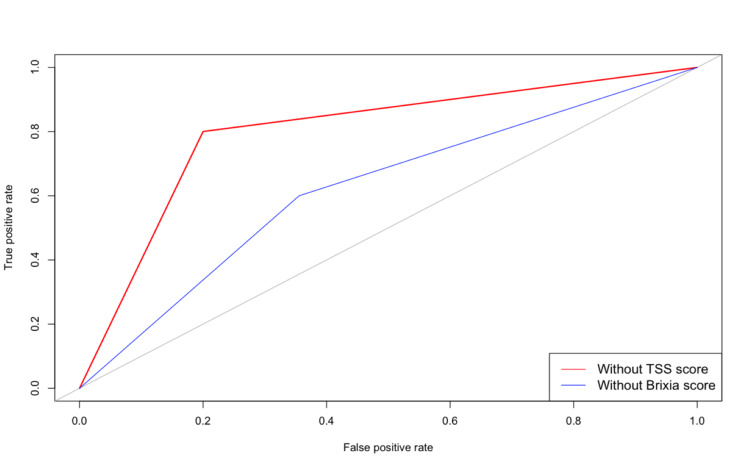
Area under the curve for the best parameter in the GBM model GBM: gradient boosting machine

A GBM was performed in the training dataset to select the best hyperparameters for predicting the mortality of patients. The best parameter based on accuracy for the included Brixia score (exclude TSS score) was 500 trees (number of iterations and number of basis functions in the additive expansion), interaction depth was 2, shrinkage was 0.01, and ﻿the minimum number of observations in the terminal nodes of the trees was 20. The test dataset's accuracy was 80.0% (95% CI: 67.0% - 89.6%) with 0.80 sensitivity and 0.80 specificity. The AUC was 0.80. The best parameter for the model included the TSS score (excluding the Brixia score) and was the same as the above model. However, the accuracy was 63.6% (95% CI: 49.6% - 76.2%) with 0.60 sensitivity and 0.64 specificity. The area under the curve was 0.62. The receiver operating characteristic curve for both models is shown in Figure 4.

## Discussion

In adult patients with COVID-19, much research reveals that older people with underlying comorbidities (such as hypertension, diabetes, cardiovascular disease, and oncologic history) have the worst prognosis. They most often end up with worse outcomes such as acute respiratory distress syndrome (ARDS) and pneumonia [12-13,16,20-21]. In our study, the median age was 59 years, and the age of the death group (64.8 ± 12.8) was higher than the living group (58.5 ± 13.9); the difference was statistically significant with p = 0.003. Furthermore, the death group has a higher BMI, comorbidity rate, NEWS2 score, and contains predominantly women. According to the univariate and multivariate analyses, previous comorbidity was the highest prognostic indicator of death outcome.

Imaging also plays a role in the predictive assessment and patient stratification in COVID-19 pneumonia. During this pandemic, in field hospitals, due to easy availability, faster results, less radiation exposure, easy disinfection procedure, and minimum risk of cross-infection, portable CXR plays a non-inferior role for an accurate radiological approach than chest CT-scan [16,20]. To the best of our knowledge, this study is the first study to examine the relationship between CXR severity scores and all-cause mortality in COVID-19 patients who presented early to a Vietnamese primary health care center. Some CXR scoring systems have recently been developed as a semi-quantitative tool for evaluating lung abnormalities providing valuable help to clinicians, and improving the stratification of the disease's risk [6-7]. In particular, higher disease scores at baseline have been associated with hospitalization, a requirement for mechanical ventilation, and in-hospital mortality [6,21]. Nava-Munoz found that initial CXR can be helpful to use a scale of radiological severity to classify chest X-rays [14]. The more significant the radiological severity or need for hospitalization, the more significant the alteration of laboratory parameters would be [14]. Our study compares the Brixia and TSS scores studied more extensively in tertiary hospitals. Our results showed that the Brixia score is superior to the TSS score in predicting in-hospital mortality. While TSS scores have a relatively small association with recovery rate, the Brixia score has been ranked the most important, with the highest SHAP value.

Moreover, the Brixia score positively correlated with other clinical indicators such as BMI, NEWS2, and age. The Brixia score improved clinical decision-making, especially in COVID-19 patients with moderate-to-severe signs and symptoms. The Brixia score should be included in a predictive model with other clinical factors. Both scoring systems have been demonstrated to be valuably prognostic in predicting ICU admission and mortality in resource-constrained scenarios [22-24]. Compared to the Brixia score, the TSS score is quick and straightforward to calculate, reflected in its calculation speed. Thus, the Brixia score may be more difficult to understand for junior medical staff, and increased complexity may add to existing pressures on staff given the increasingly recognized risks of burnout. These pressures could be an obstacle for primary healthcare physicians who require an accurate and straightforward technique for the early diagnosis of COVID-19 pneumonia but lack the knowledge and experience to do so. A modified CXR scoring system designed by Dr. Soetomo, adopted from the Brixia score and TSS score, has a similar correlation with the clinical severity of the disease [25-26]. Further study must be carried out to validate the modified score in the Vietnamese population.

Although we did not analyze the follow-up chest X-ray score with the mortality of COVID-19 patients, future studies could determine whether there is a correlation between the radiological and clinical progression of the disease. This study highlights the role that CXR scoring can play in secondary/primary-level hospitals where the high number of case workloads can overwhelm a healthcare system, especially in countries with a weak public healthcare system combined with a lower socioeconomic structure. CXR could then be used to assess which patients may need transfer to tertiary care, especially in countries with a surge of cases where healthcare is stretched to a tipping point. Moreover, we have to highlight the potential role of routine clinical parameters integrated with a portable CXR scoring system, a low-cost and primarily available technique, to stratify infected patients according to their risk of worsening. These considerations might have clinical relevance in countries with limited resources.

Our study has several limitations. First, this study lacks standard reference imaging for comparison, as chest CT was not performed routinely in COVID-19 patients at our institution, which could have led to chest X-rays with undetected opacities. Second, since it was a single-center retrospective study, all patients were positive for RT-PCR (clinical diagnoses of SARS-CoV-2 were not included). Only admitted patients who underwent chest radiography were studied, which can limit the generalizability of our results, particularly to mild cases. Another limitation was the small sample size, which is due to the nature of the study. Therefore, our results require confirmation with a larger cohort of patients and performance in different hospitals with various validation conditions. Furthermore, the participating radiologists were not all chest radiologists but were all highly experienced in chest radiograph reporting, especially during the pandemic, thus reflecting real-world practice.

## Conclusions

In conclusion, at the peak of the epidemic, especially in areas with resource and specialist shortages, applying a radiological score through portable CXR imaging helps clinicians measure the severity of COVID-19 and have more effective patient management. CXRs are a good monitoring tool for COVID-19 chest manifestations, and their scoring system provides an accurate method of predicting disease severity. The Brixia score is strongly correlated with the severity and outcome of the diseases. This study suggested that the Brixia score and NEWS2 could be incorporated into a predictive model to predict in-hospital mortality, essentially in Vietnamese field hospitals.
